# Medical Society of the County of Erie

**Published:** 1893-08

**Authors:** Eli H. Long

**Affiliations:** Secretary


### Society Proceedings.

### MEDICAL SOCIETY OF THE COUNTY OF ERIE.

### SEMI-ANNUAL MEETING, JUNE 13, 1893.

Reported by ELI H. LONG, M. D., Secretary.

MEETING called to order in the rooms of the Buffalo Academy of Medicine, at 10.45 A. M., by the President, DR. JOHN PARMENTER.

The minutes of the annual meeting and of the special meeting of April 20th, were read and adopted.

The Committee on Membership reported favorably upon the following-named physicians, and upon motion, duly made and seconded, they were unanimously elected to membership: Albert F. Erb, Edward L. Frost, Franklin C. Gram, George J. Hearne, Geo. A. Himmelsbach, H. Corwin Jones, Chas. E. Long, Edward J. Meyer, Ferdinand G. Mochlan, Duncan Sinclair, James Stoddart, Clarence A. Tyler, G. W. Wende, J. F. Whitwell, Edward R. Wiser.

The committee could not report upon the following, as they had not yet exhibited their diplomas to the committee: Charles H. Perry, Francis Metcalfe, Cyrus S. Siegfried.

Applications by the following-named physicians for membership in the society were read and referred to the Membership Committee: A. W. Bayliss, Horace Clark, Francis A. Drake, Chas. S. Jewett, Ada C. Latham, Albert T. Lytle, Harry Mead, DeWitt H. Sherman.

The President made an informal report concerning the library, to the effect that progress was being made in locating the same in the new library of the University of Buffalo.

The committee on revision of the by-laws, appointed at the special meeting, held April 20th, presented and read the proposed amendments, which, according to rule, were held for action at the next regular meeting.

Communications were read from the Medical Society of the State of New York, reporting list of officers, chairmen of committees, etc., and from the Central New York Medical Association, announcing the next annual meeting to be held June 16th, in Rochester. The resignation of Dr. P. W. VanPeyma, from the Board of Censors, was read, and upon motion having been made and seconded, the same was accepted.

The Secretary moved the matter of filling the vacancy upon the Board of Censors be postponed until the regular time for election. Carried.

It was moved and seconded that an order be drawn for the sum of $20.00, in favor of Dr. J. F. Krug, to reimburse him for payment of bill for legal services required by the Board of Censors. Carried.

The Society then proceeded to the scientific part of the day's programme.

The discussion on the Use of the Metric System was introduced by the report of the Committee on the Metric System. Dr. Benedict, as chairman, reported at some length in reference to the extension of the use of the metric system in foreign countries. He also presented the following resolutions, which, after discussion by Drs. Rochester, Long, and Benedict, were adopted:

### RESOLUTIONS.

WHEREAS—We, the members of the Medical Society of the County of Erie, believe that the increasing use of the metric system by physicians, warrants a more formal recognition than has hitherto been accorded; and

WHEREAS—We believe that the metric system, in addition to the theoretical advantages common to all decimal systems of denominate numbers, provides units of practical size and conveniently correlated.

*Resolved*—That we recommend the use of the metric system to physicians and pharmacists in particular, and that, to render its general employment feasible, we approve of the thorough teaching of this system in educational institutions, both professional and academic ; and

*Resolved*—That copies of these resolutions be transmitted to the President of the Erie County Pharmaceutical Association, the Superintendent of Education of the City of Buffalo, the University of Buffalo, the Medical Department of Niagara University, and the BUFFALO MEDICAL AND SURGICAL JOURNAL.

At 12.15 a recess was taken until 3 P. M.

### AFTERNOON SESSION.

The society was called to order at 3.15 by the President.

The following memorial of William D. Murray, M. D., was read by Dr. R. C. TABER, of Tonawanda :

Dr. William D. Murray was born near Lockport, Niagara county, N. Y., September 1, 1833, and died at Tonawanda, February 23, 1893, a span of three score years, lacking six months. He was reared on his father's farm, and as a school boy walked to and from the Lockport school, a distance of four miles, rather than content himself with the limited advantages offered nearer home.

Later, we find him devoting the winter months to school-teaching, returning to the farm work during the summer season.

He early decided upon the practice of medicine for his life-work, and entered the office of Drs. Fassett and Kittinger, at Lockport. Actuated by a persistent spirit and a love to gratify his work-habit, he became a close student and observer. At the age of twenty-six years, armed with the degree of M. D., conferred by the Columbia Medical College, of Washington, he started out in his career—practicing medicine in Tonawanda, Erie county.

In 1861, he was commissioned Assistant-Surgeon of the 100th New York Volunteers, serving honorably under his old preceptor, Dr. Kittinger, until his discharge, after which he returned to Tonawanda, to practice more effectively with the knowledge thus acquired in this practical school. His career was marked by a constant application of his best efforts, always at work, never lured from his path by glittering promises in other fields, backed up by a robust physique and a willingness to do, he soon found himself in the list of successful practitioners, a representative citizen, in touch with all classes and interests, one whose presence is felt in the community and in the councils of his townsmen, as well as at the bedside.

His efficient work in the Educational Board of his native town has been appropriately commemorated by giving his name to the beautiful school building which has grown up under his administration.

He was one of the original members of the New York Medical Association. He became a member of this society in 18—, and he will be remembered as one who took an active part in the councils which brought forth the Fee List of 1871.

At the time of his death he was a member of the Masonic fraternity, a very active member of the Grand Army, and Surgeon of the 25th Separate Company of the National Guard.

But the sturdy, robust, and genial worker is gone, and we are again reminded of the limitations of our art, and admonished to so conduct our affairs, that when the hour strikes for us, we will find placed to our credit as many virtues as were possessed by our late fellow, Dr. Murray.

Moved that the memorial be received and incorporated in the minutes. Carried.

Dr. Irving M. Snow then read a paper upon A Gastric Neurosis in Childhood. The paper, with reported cases, proved to be of great interest to those present, and discussion was freely participated in.

Dr. A. A. Hubbell asked consent to introduce a motion at this time, to the effect that Drs. Benedict, Crego, and Thornbury be elected delegates to the Central New York Medical Association, in addition to those who had been appointed. Consent was given, and the motion was carried unanimously.

The remainder of the programme consisted of the following papers upon Renal Insufficiency: Diagnosis, by Dr. Allan A. Jones; Treatment, by Dr. DeLancey Rochester. The papers were regarded as possessing special merit, and the subject was further discussed by Drs. Hayd, Bartlett, Pryor, and Long.

Dr. Rochester moved that the secretary be authorized to issue proofs of the By-laws, as reported by the Committee on Revision, for use of the members at the next regular meeting. Carried.

Adjourned at 5.40 P. M.

---

Parasitic Origin of Cancer.—The facts showing that there are in the cells of cancerous tumors small parasitic bodies of the nature of protozoa, are continually accumulating. Dr. James Galloway, in the Morton Lectures, reports the results of his studies of the life history of analogous organisms in rabbits. He confirms the statements of Rauffer and others, to the effect that the peculiar protozoa of cancer may be found in all the tumors.

